# Evidence-Based Practice in Psychosocial Oncology from the Perspective of Canadian Service Directors

**DOI:** 10.3390/curroncol30040303

**Published:** 2023-04-01

**Authors:** Sarah Mackay, Viviane Ta, Sebastien Dewez, Annett Körner

**Affiliations:** 1 Department of Educational & Counselling Psychology, McGill University, 3700 McTavish St, Montreal, QC H3A 1Y2, Canada; 2 Department of Psychology, University of Montreal, 90 Avenue Vincent d’Indy, Montreal, QC H2V 2S9, Canada; 3 Lady Davis Institute, Jewish General Hospital, 3755 Chemin de la Côte-Sainte-Catherine, Montreal, QC H3T 1E2, Canada; 4 Department of Oncology, McGill University, 5100 de Maisonneuve Blvd. West, Suite 720, Montreal, QC H4A 3T2, Canada; 5 Louise Granofsky Psychosocial Oncology Program, Segal Cancer Centre, 3755 Côte-Sainte-Catherine Road, Montreal, QC H3T 1E2, Canada; 6 Psychosocial Oncology Program, McGill University Health Centre, 1001 Décarie Blvd, Room D02.9005, Montreal, QC H4A 3J1, Canada

**Keywords:** cancer, evidence-based practice, psychosocial oncology, psycho-oncology, Canada

## Abstract

Evidence-based practices facilitate the effective delivery of psychological services, yet research on the implementation of evidence-based practices in psychosocial oncology (PSO) is scarce. Responding to this gap, we interviewed a diverse sample of 16 directors of Canadian psychosocial oncology services about (a) how evidence-based practices in psychosocial oncology are being implemented in clinical care and how the service quality is monitored and (b) what are barriers and facilitators to evidence-based practice in psychosocial oncology services? Responses were grouped according to three main themes emerging from the data: screening for distress and referral to PSO services, delivery of evidence-based PSO services, and monitoring of PSO services. Our findings highlight facilitators and barriers to evidence-based practice in psychosocial oncology, which were related to the political, social, economic, and geographic contexts. The stepped care model was identified as a science-informed approach to improve the cost-effectiveness of triage systems and treatment delivery while facilitating more equitable access to services. Other facilitators included electronic screening and referral systems as well as protected time for clinicians to communicate more within their teams and participate in knowledge exchange. High caseloads presented a major barrier to acquiring and implementing evidence-based practices. Recommen–dations include increased support for evidence-based onboarding and continued training as well as for data collection regarding service needs, quality, and quantity to inform service monitoring and advocacy for more financial resources. Our findings are relevant to healthcare decision makers, implementation researchers, as well as service directors and practitioners providing psychosocial oncology care.

## 1. Introduction

### 1.1. Introduction to Evidence-Based Practice

Regulatory bodies in psychology require professional training and clinical practice to be based on scientific evidence [1,2]. In addition, third-party payers are increasingly demanding outcome-driven, cost-effective care models [3] (p. 204). Science-informed practice refers to using the latest, best-available scientific evidence to inform all aspects of clinical service delivery. Evidence-based practice (EBP) as defined by the American and the Canadian Psychological Associations goes a step further by prescribing how science is to be used to guide clinical practice, i.e., through the conscientious integration of three fundamental components of EBP: (1) best scientific evidence, (2) clinical expertise, and (3) characteristics, treatment preferences, and cultural background of the patient [4,5,6,7]. EBP further entails the monitoring of treatment outcomes from intake to termination to inform treatment planning, modification, and discontinuation. Implied in the definition of EBP is a commitment to continuously being informed by the best scientific evidence. However, clinicians frequently report insufficient time or resources for developing and maintaining a working knowledge of the latest scientific evidence, which is exacerbated by an ever-increasing demand for mental health services [8,9].

### 1.2. Evidence-Based Practice in Psychosocial Oncology

Patients and their social support systems have mental health needs that require attention across the cancer trajectory [10]. A wealth of research has shown the effectiveness and value of addressing these needs through psychosocial oncology (PSO) interventions in routine cancer care [11,12]. Yet, despite increased awareness and demonstrated value of PSO care, patients report that their psychosocial struggles and symptoms are often not sufficiently addressed and sometimes not even acknowledged [13]. Some individual and systemic barriers to EBP in PSO have been mentioned in the literature, such as high patient-to-clinician ratios or an overwhelming amount of relevant scientific literature [14,15].

Over the past decade, evidence summaries and clinical decision-making resources, such as clinical practice guidelines, have been created to facilitate EBP in PSO throughout the cancer trajectory [13]. Clinical practice guidelines systematically evaluate and summarize the latest scientific evidence for specific symptoms or conditions and synthesize findings into actionable recommendations for clinical practice. Given the growing knowledge base on psychological interventions specific to PSO, guidelines have been developed that provide clinicians with evidence-based recommendations, e.g., for screening, assessment, and treatment of cancer-related distress, depression, fear of cancer recurrence, and for psychosocial support of cancer survivors [11,16,17]. Researchers have noted that there are extensive variations in the usage of clinical practice guidelines, pointing out challenges such as insufficient research on the complex processes of guideline implementation, difficulty in keeping guidelines up to date with the newest evidence, knowledge and attitude barriers at the clinician level, patient concerns about the stigma of needing counselling, as well as resource barriers at the systemic level [18].

### 1.3. Research Objectives

To our knowledge, there is no empirical evidence detailing the use of EBP in PSO services provided to adult patients. Wiener et al. (2015) published the only recent study that examined the state of EBP in pediatric PSO, focusing specifically on children with cancer and their families [3]. The current study examines the perspective of Canadian directors, coordinators, and managers of PSO services regarding evidence-based psychosocial care for adults (18+) diagnosed with cancer and their families. Our research questions are as follows: (1) how are evidence-based practices in psychosocial oncology being implemented in clinical care and how is the service quality monitored, and (2) what are the barriers and facilitators of evidence-based practices in Canadian psychosocial oncology services?

## 2. Materials and Methods

### 2.1. Research Design Overview

This study presents primary data based on semi-structured phone interviews with directors, managers, and coordinators of PSO services in Canada. Reflexive notetaking was used as a secondary data collection strategy. The qualitative data-analytic procedure incorporated inductive and deductive generation of themes in an emerging process. The approach to inquiry and philosophical assumptions underpinning this research design are rooted in a social constructivist paradigm. Knowledge was understood to be co-constructed through dynamic interactions between participants and researchers [19,20].

As primary investigator of this project, S.M. complemented their graduate training in qualitative inquiry by consulting two qualitative experts and participating in monthly meetings of McGill’s Qualitative Health Research group as part of their commitment to broadening the scope of their qualitative method knowledge base and expertise [21]. Their understanding of the phenomena under study has been informed by an in-depth literature review that influenced the structure of data collection and the lens through which data analysis occurred. To manage this influence, S.M. used a bottom-up approach to thematic analysis and two additional independent coders helped develop the coding manual and analyze data. S.M.’s interest in conducting this study was motivated by understanding the complexities of psychology as a discipline and how Canadian healthcare standards and contextual factors continuously shape program structures. Finally, S.M. had no prior relationship with any of the study participants.

### 2.2. Procedures and Participants

The Research Ethics Board of McGill University granted ethical approval for this project (#104-0719). Purposeful snowball sampling, whereby participants may refer additional respondents, was the primary recruitment method due to its practical advantage in accessing a network of professionals who are few in number [22]. The distribution of participants resulted from objective probability and deliberate selection. An online advertisement was posted in the monthly newsletter of the Canadian Association of Psychosocial Oncology. Researchers also shared the advertisement with PSO services identified though online searches to ensure the inclusion of service providers from various provinces and to reach potential interviewees from geographical regions that had not yet been represented in the study sample. Healthcare professionals were eligible to participate if they were clinical directors, managers, or coordinators of PSO services provided within hospitals, cancer centres, or community-based institutions, but not private practices, in Canada.

The interview guide was co-constructed in consultation with PSO experts (please see Supplementary Material for the interview guide). Participants were asked open-ended questions about their program’s structure; about their current policies and standards of care as it relates to EBP; barriers and facilitators for the delivery of EBP; and details concerning research access and ongoing education opportunities for clinicians. Common participant questions as well as the evolving findings led to minor alterations in the interview guide. Individual interviews were audio-recorded, transcribed verbatim, and anonymized. Transcripts, field notes, and debrief discussion notes were used for analyses. Initial data analyses were conducted alongside data collection. The interviews ranged from 45 to 82 min ($\bar{X}$ = 62.3 min) and were completed between November 2019 and June 2020. Recruitment was terminated when authors agreed that thematic saturation had been achieved.

### 2.3. Data Analyses

Thematic data analysis was conducted following Braun and Clarke’s (2006) guidelines for immersion in the data, generating initial codes, as well as reviewing and defining simply the fewest number of content themes [23]. S.M. used NVivo software 12.0 and noted initial codes and emerging content themes. The coding team read transcripts focusing on participant descriptions of the implementation of EBP in PSO, monitoring PSO service quality, as well as the barriers and facilitators to this. S.M. and V.T. independently coded two transcripts and compared their respective coded text segments according to a coding manual. Discrepancies in coding and definitions of codes were discussed until consensus was reached with assistance from A.K. when necessary. Consensus discussions led to final modifications of the coding scheme and consolidated a common understanding of the codes between coders.

Intercoder reliability (ICR) was calculated using the Mezzich procedure [24,25] by applying 44 non-mutually exclusive codes to 4 interview transcripts coded independently by S.M. and V.T (please see Supplementary Material for the Intercoder Reliability Data Table). Two selection criteria were used in the assessment of ICR: codes had to address substantive issues related to the research questions and needed to appear with reasonable frequency of at least three times in the text [26]. Concordances and discordances were listed in a cross-table and regarded as concordant if both coders assigned the main statement of the text to the same code. The overall Mezzich’s kappa coefficient for the 32 codes was 0.64, which indicates significant agreement at *t*(31) = 5.66, *p* < 0.001, i.e., a moderate level of agreement [27]. Intercoder agreement (ICA) on any discordances was 99.6%. S.M. coded the remaining 9 transcripts independently.

## 3. Results

In total, sixteen directors from thirteen unique clinical sites participated in this study (five hospitals, six cancer centres, two community centres), the majority of which were university affiliated (69%). Hospital sites were most likely to be affiliated to a university (100%), followed by cancer centres (67%), while no community centres were affiliated with a university. Table 1 provides a detailed overview of the sociodemographic characteristics of the study sample. The sample was diverse in terms of gender (62% women), age (44% between 45–54 years), educational attainment, and years working in the field of PSO (range: 1–38 years, $\bar{X}$ = 10.4 years, SD = 9.8). The sample was more homogenous in terms of their field of training (38% social work, 31% psychology) and geographical location (31% Ontario, 25% Quebec, 44% other Canadian provinces). The professions of study participants, in addition to social work and psychology, also included nursing, occupational therapy, kinesiology, and psychiatry.

Interview results are presented according to the two research questions (please see Table 2 for a brief overview of the themes identified through qualitative analysis). In the interest of brevity, the researchers tell the story of the participants, keeping quotes to a minimum, which have been lightly edited for conciseness and clarity while retaining their original meaning.

**Findings regarding research question 1: How are evidence-based practices in psychosocial oncology implemented in clinical care and how is the service quality monitored?**

### 3.1. Screening for Distress and Referral to PSO Services

#### 3.1.1. Initial and Repeated Distress Screening

Few topics received as much attention as the implementation of distress screening because of its importance in accurately identifying patients in need of PSO care. One participant noted that patients differ in their expression of distress in saying: “they’re looking stoic and brave but aren’t referred because they’re not crying in the office”. All participants except for one described some variation in initial distress screening during patients’ first visit, with high scores prompting further exploration. The use of diverse screening instruments was mentioned, such as the Patient Health Questionnaire (PHQ-9), the Generalized Anxiety Disorder Scale (GAD-7), the Canadian Problem Checklist from the Canadian Partnership Against Cancer (CPAC), and the Distress Thermometer. Participants expressed that repeated distress screening, which would trigger a PSO referral whenever indicated across the cancer trajectory, was inconsistently and rarely implemented.

Some participants detailed discrepancies between their intended operating procedures and the reality of initial distress screening. The participant, who disclosed their program had no formalized distress screening, indicated they missed patients in need while other patients were viewed by more than one clinician. Another participant shared that frontline healthcare workers omitted the initial distress screening when they knew that sufficient follow-up PSO services would not be available. Notably, one participant commented that patients reporting distress higher than two on a ten-point scale should receive follow-up assessment, but follow-up was only happening for patients with scores above seven. They added that, according to certain stakeholders, initial oncology appointments are often so loaded with information that psychosocial needs are the last to be addressed; by that time, patients feel too overwhelmed to engage in psychosocial assessment.

#### 3.1.2. Triage and Referral Systems

Participants also highlighted the importance of triage and referral systems to connect cancer patients in distress with appropriate PSO services. Some programs had only the most basic referral system, while others reported high levels of multidisciplinary, departmental, and external cooperation. One participant described triaging according to the stepped care model, where symptom management and group programming are offered first, followed by more individualized care for those for whom this is not effective in managing their distress. It was expressed that programs have a responsibility to (a) have a working knowledge of internal and external resources available to patients as well as their acceptance criteria, and (b) visibly promote available services and ensure patients are informed about available services in a timely manner to maximize service uptake. Some sites emphasized the importance of patients being able to express their treatment preferences. 

Several challenges to these perceived responsibilities were described. Participants expressed that it is difficult for clinicians to maintain a working knowledge of resources due to the frequently changing availability and acceptance criteria of PSO services. One participant explained that their province-wide fatigue management group was poorly attended due to low awareness of its availability among healthcare providers and patients where it might have otherwise been beneficial since cancer patients commonly suffer from fatigue. Moreover, when discussing recent survey findings indicating that patients did not know about services soon enough, one participant questioned: “whether or not patients were told; if they recalled; or if the timing was right”.

#### 3.1.3. Administration and Technology

Some participants explained that, for efficiency reasons, their PSO programs created designated positions, such as administrative assistants and resource counsellors. This decreases the burden for each individual clinician and frontline staff to stay up to date with all available internal and external resources, including the respective eligibility criteria. Staff in these roles either helped or completely managed initial and repeated distress screening as well as the triage and referral processes. These roles were also supportive in that they could provide patient orientation, update and organizing service guides with programming options, and opportunities for patients to express treatment preferences before starting a specific service. One participant exemplified the usefulness of such administrative support:

“Our program has upwards of about 2500 h of programming we offer a month. So participants are assigned a wellness guide with all the different programs that they can take which will follow them… So, we periodically call in, see how they’re doing, see what they like, what they don’t like, what their needs are… Every month a new calendar comes out and they select their wish list, and a team actually assigns each participant based on their wishes and based on our availability for that month.”

An electronic referral system was also emphasized as a key facilitative technology for the implementation of distress screening, whereby patients complete screening tools on a tablet or kiosk. In this PSO program, staff were then automatically notified to follow up with patients based on predetermined cut-off scores. This reportedly led to higher rates of patients receiving PSO care than when screening was conducted manually by oncologists and nurses. Other facilitative technologies included digitizing case management and using a secure online platform to make files accessible to clinicians and patients.

### 3.2. Delivery of Evidence-Based PSO Services

#### 3.2.1. Therapeutic Interventions

Some participants described using the stepped care model to guide the implementation of their PSO services, where symptom management and group programming are offered first, followed by more individualized care for those for whom this is not effective in managing their distress. Some also noted that, prior to implementing new group programming, scientific evidence for its efficacy had to be provided to healthcare decision makers external to the PSO program. Finally, participants also remarked that offering group programming and training others to offer this is a specialized skillset that is not easily replaced: “We have a clinical nurse specialist who resigned—she was the one who did all the training around the physical symptoms and side effects of cancer with women in particular”.

Participants described that clinicians used approaches specifically developed and validated in the cancer context, including CALM therapy, dignity therapy, and meaning-centered therapy. They also mentioned other evidence-based approaches commonly used in mental health disciplines, such as cognitive behavioural therapy, mindfulness-based interventions, motivational interviewing, and narrative therapy. Most participants indicated that these services are offered in an integrative fashion, i.e., adapted to the unique needs of each patient.

#### 3.2.2. Credentials and Prior Professional Experience

Participants specified requirements in PSO programs’ hiring processes, such as the expectation for clinicians to have pre-existing skillsets related to EBP in PSO. Except for administrative assistants or resource counsellors, clinicians were expected to have a relevant master’s degree and discipline-specific licensure. Moreover, participants commented that clinicians with experience in the healthcare system were viewed more favorably in the hiring process due to likely having transferrable knowledge and skills. They further expressed that experience in oncology-related areas, such as chronic illness or palliative care, helped clinicians to better understand medical treatments in oncology and the unique physical and psychological side effects associated with cancer. As one participant put it: “Offering evidence-based treatment requires that a clinician understands the cancer trajectory and related issues like treatments, side effects, and fatigue.” If clinicians did not already arrive with oncology-specific expertise and experience, it was expected that they would gradually develop it.

#### 3.2.3. Onboarding Training Protocols

Onboarding training protocols can be understood as a transition point between clinicians’ prior professional experiences and the need to acquire and maintain knowledge regarding EBP in PSO. Most participants described that their PSO program had at least an informal training protocol that was intended to guide new staff members and student clinicians in the delivery of services. Informal training sometimes included a shadowing or mentorship period, orientation to PSO care, administrative orientation, and recommended readings. Fewer participants described their PSO program had a comprehensive and periodically reviewed training protocol to facilitate EBP. These protocols included, for example, ongoing supervisory support, access to online training courses, and opportunities for specialist training. One participant emphasized that their PSO program’s training procedures are informed by synthesized scientific evidence, whereas another participant shared that, as a program director, it is challenging to create training protocols incorporating the latest research evidence: “From a manager’s perspective, I find that there’s no help with developing those protocols”.

#### 3.2.4. Ongoing Access to Research and Training

Participants described that ongoing access to primary research and research summaries were important for the maintenance of EBP in PSO services. Participants remarked that clinicians relied more on merged evidence resources rather than on original research studies when deciding how to implement interventions. Examples of sources at the national level included the clinical practice guidelines from the Canadian Association of Psychosocial Oncology and distance education opportunities from the Interprofessional Psychosocial Oncology Distance Education, as well as provincial guidelines from organizations such as Cancer Care Ontario.

Participants noted two main barriers to remaining science-informed. The first was a lack of access to scientific databases such as those available at university libraries, which one participant circumvented by accessing others’ library access but expressed considerable concern:

“One of the largest barriers for us is that individual clinicians, including me as clinical lead and our manager, no longer have direct access to the university library system—they deleted our library. So, if we want to do searches in the library, you have to by hook or by crook, find somebody who has access. I used to do it with my daughter, but she doesn’t go to university anymore. So, I can’t look up or follow up on something unless I jump through hoops […] Well, you know that’s not good enough. So that’s a big deal. That’s a real barrier”.

Other participants highlighted that the maintenance of PSO knowledge requires a considerable amount of time and effort on the part of clinicians, as one participant noted:

“You can go to a seminar, but the real challenge is that when you go back to your normal life, where you work—how much time do you have for implementation, follow-up, and support to become more proficient with whatever it is that you’ve learned? Sometimes I think that getting training is half the battle: the other half is maintaining the resolve and protecting the time, finding your own internal motivation and external support to continue to use it”.

Participants reported cooperative sharing, which refers to sharing knowledge of EBP within the PSO community through collegial interactions. This fostered clinicians’ maintenance of their EBP knowledge in PSO. Activities exemplifying cooperative sharing included supervision and mentorship, journal clubs and communities of practice, peer and case consultations, specialist presentations via visiting speakers, conferences or symposiums, and research collaboration. While participants felt that advanced scientific literacy was associated with doctoral degrees, it was also expressed that scientific literacy among clinicians could be facilitated through discussions of the merits and limitations of PSO research. Along these lines, the second barrier to consuming research was a universally voiced concern about having too little time for all the activities that would enable clinicians to remain science-informed. This is elaborated further under political context.

#### 3.2.5. Clinician Attitude and Specialization

Participants reported that facilitators of EBP included clinicians’ commitment to ongoing learning and self-reflection as well as having chosen to specialize further within PSO, e.g., disease/tumor site, population attributes, or therapeutic approach. Ongoing learning was said to improve patient care but also foster clinician wellbeing. As one participant put it: “I don’t think, if you’re just seeing patients hour after hour that sense of openness to new ideas is there. I’ve done best when I see cancer patients and when I’m actively involved in research—I feel it keeps me interested, fresh”. Specialization was universally described to be of benefit to patients, whose unique needs might be better addressed through tailored care, as well as to clinicians, and more broadly to the field of PSO. Narrowing one’s clinical focus led to more relevant patient referrals for the clinician, made it easier for the clinician to keep up with research in a specific area, increased their confidence, and also helped to inform research agendas.

### 3.3. Monitoring of PSO Services

Participants described three areas of monitoring needed: at the patient level, that is, if are patients reporting that the PSO services are effective in managing and reducing distress; at the clinician level, which procedures are in place to ensure services are evidence-based; and at the program level, what the overall outcomes of services provided are at a given PSO site.

#### 3.3.1. Patient Feedback

Participants expressed that patient feedback could be acquired through repeated distress screening, patient satisfaction surveys, verbal feedback, and by welcoming patient advisory groups. One participant noted the importance of explicitly soliciting patient feedback, explaining that, while patients rarely complained about their care, they are not necessarily given the opportunity to share their concerns about what is or is not working. Outcome monitoring via patient feedback was described as instrumental for improving the quality of PSO care as it informed the science sought out by clinicians to gain additional knowledge. Moreover, when PSO programs were able to gather these data, it also helped justifying resource acquisition from health authorities and expansions in programming to increase the quantity of care offered. However, some participants expressed concerns that, even though patient feedback was solicited, little was done with this data, either because the data were not specific to their PSO program and/or because they had too little time to act on it. One participant shared:

“[Provincial Cancer Care Organization], requires that patients complete their ESAS [Edmonton Symptom Assessment System] every once and a while. We do get those results back but they’re not specific to the program that you’re in or just for the psychosocial oncology program—we’d be getting results for the [oncology] program as a whole. So, it’s hard to use that data within our program or to evaluate what we’re actually doing”.

#### 3.3.2. Requirements of Licensing Bodies and Performance Reviews

Participants explained that, at the individual clinician level, they needed to trust regulatory colleges to monitor that their members adhere to their respective standards of practice and work within their scope in an evidence-based manner. Colleges handle this largely through continuing education requirements. Certain participants expressed monitoring at PSO programs occurred through annual audits or performance reviews, which allowed them to substantiate whether clinicians were offering evidence-based services. Participants added that these were useful opportunities to provide constructive feedback to clinicians and collaboratively set professional goals for upcoming periods.

#### 3.3.3. Program Evaluation

Many participants reported that their PSO program had some kind of program-wide monitoring, even if this was completed informally and infrequently. Most programs collected productivity statistics requested by health authorities, such as patient wait times, number of patients served and each clinician’s caseload. Participants universally felt there was a distinct emphasis on quantity of care and explained that the primary reason for not collecting program-wide patient outcome data was due to health authorities almost exclusively allocating resources for documenting the number of patients seen. Participants had recommendations for important data on service quality to be gathered (besides the patient-reported outcomes already mentioned under *Patient Feedback*), e.g., tracking the number of patients who accepted referrals to external resources when internal ones could not be offered and monitoring the uptake of group programming relative to spaces available in a group. 

#### 3.3.4. Research and Quality Assurance Projects

Participants described research and quality assurance projects that included obtaining and reviewing patient feedback and completing program evaluations. They stated that such projects held the potential to maximize the use of existing resources and helped advocate for the expansion of PSO services. Other participants described how being involved in research or quality assurance projects helped improve the effectiveness of services:

“We were able to carry out clinical research, very interesting projects, with young adults with cancer with specific symptom management projects around brachytherapy for colorectal cancer. There were papers published based on this data. So, we had research as a way of keeping us at the edge. The science practitioner model is the best way for clinicians to hone their skills because they have to review the literature, they have to know what’s going on—you’re examining interventions and programs and help service delivery models that bring better care to patients”.

However, participants explained that, if clinicians were permitted to be involved in research projects, they tended to be collaborators. Some participants explained this was due to time constraints and not having research mandates in their job descriptions. Participants expressed concern that, even at institutes where research is mandated, the support for such projects was inconsistent: “We’re in a teaching hospital of [a large university] so part of our mandate was to do clinical research. When the ministry changed, research became less of a priority [and stopped being given] real support”. As such, research was most often led by external primary investigators while PSO clinicians were involved as collaborators or as research participants”.

**Findings regarding research question 2: What are the barriers and facilitators of evidence-based practice in psychosocial oncology services?**

Barriers and facilitators were described along four different but overlapping contexts: political, social, economic, and geographical. These factors contributed to the unique situation of each PSO program and by extension to the way the programs implemented and monitored EBP.

### 3.4. Political Context

The term *health authority* was brought up when alluding to or specifically discussing power differentials that occurred between organizations and programs (political context) as well as directors and clinicians (social context) positioned within a vertical hierarchy. External health authorities included bodies such as the federal Public Health Agency of Canada, provincial Ministries of Health, as well as provincial cancer care organizations. Internal health authorities included PSO program leaders who oversee clinical services (and research activities, if any) within each PSO program as well as healthcare decision makers within the organization but external to the PSO program, e.g., directors of the department of oncology, institutional managers. These political and social contexts had profound implications for the economic context of each individual PSO program, where health authorities largely had control over the resources distributed to the PSO program as well as how those resources were used. Participants explained that funds were passed through vertical hierarchies from Ministries of Health to cancer care organizations, who then distribute funds with budgeting mandates or certain directives to PSO programs. Some program directors were granted an extension of this control over resources, which is discussed further under *Social Context*.

Participants overwhelmingly shared the belief that health authorities overvalued quantity of care—that is, were more concerned with minimizing costs and maximizing volume—which created barriers for PSO programs and clinicians implementing and monitoring services. Participants reported a relative devaluation of PSO services when compared to medical treatments, noting that the latter generated more revenue and extended patient life. Participants implied that the perceptions health authorities have regarding the value of mental health services and their role in fostering patients’ quality of life was a political issue. While this may be stating the obvious, one participant expressed that: “For a PSO program to exist, the people, the health authorities, and the government— they have to believe in what we do. If there’s no belief, it’s hard to sustain our services”. Participants said that the devaluation or even systematic disincentivization of PSO services has historically caused high patient-to-clinician ratios and lack of support for activities related to quality improvement, such as continuing education and service monitoring projects.

Given that a clinician’s caseload was also a primary performance metric measured by health authorities, participants said and insinuated that taking time for any task other than direct service delivery reduced time available to see patients, which, therefore, impacted their perceived productivity. As one participant put it, if clinicians are “invested in something other than seeing patients then it affects the numbers. And the [provincial health authority] evaluate us by the number of patients we see”. A theme among participants was that significant responsibility is placed on individual clinicians to be evidence-based rather than health authorities playing a larger role to help realize this. Consequently, PSO programs varied widely in the extent they were able to support clinicians’ continuing education, with those receiving less support expressing the sentiment that it is “almost impossible to get training in any meaningful way”. One director argued that chronically high workloads make it unrealistic or even impossible for staff to remain current with science during their working hours, let alone contemplate “what should we be doing” at the program level to increase service quality and quantity. When commenting on their program evaluations, another participant said: “The barriers are mostly structural. It would be great to have our own budget, our own team, and some control over these. That would have helped us to do the program evaluations, which we couldn’t do”. Ultimately, while inherent resource limitations do necessitate a focus on efficiency, the push to “do more with less” was echoed by all participants, who described being underfunded and understaffed despite the enormous and growing need for PSO services.

Participants highlighted the health authorities’ potential to play a greater role in monitoring PSO programs through program evaluations as well as providing directors and clinicians with greater autonomy in monitoring their own services. One participant described that their health authority asked their PSO program to choose and apply recommendations from a list, then annually report back on their progress: “[Provincial Cancer Center] came out last year with thirty recommendations considered to be standards of care. We were tasked to take three of those recommendations and apply them.” Another participant spoke of a provincial pilot to fund services based on scientific evidence for services that respond to patient needs:

“Part of our health system reform has been using a new type of funding developed by our provincial health authority. It says, “Okay, how do we implement the quality-based procedures model for radiation therapy?” and then, “What is the appropriate amount of funding for each radiation patient to ensure their psychosocial needs are met?” So, this shifts away from a fee-for-service model and instead says, “We’re going to fund your cancer centre based on what the evidence says about needs and the care that should be delivered”. So, this new model is trying to tie funding to the provision of quality services”.

Lastly, some community sites described that reduced presence of political hierarchy was helpful in servicing patients more quickly. They reported having more flexibility in managing time and finances compared to tertiary care services. This autonomy reportedly reduced bureaucracy and allowed PSO programs and clinicians to respond to patient needs in a timely manner, as explained by one participant: “The fact that we don’t have the red tape of bureaucracy helps us respond a little bit faster. There aren’t many people at our organization—so we can, for example, offer an additional cancer support group when we notice big waitlists”.

### 3.5. Social Context

#### 3.5.1. Directorial Vision

Participants described that they, as directors, are uniquely positioned to shape the implementation of PSO services, particularly when holding a leadership position over long, uninterrupted periods of time. Consequently, hiring directors specialized in PSO was emphasized for reasons having to do with strong working knowledge of PSO-specific EBP. However, participants also noted that some PSO leadership positions lacked compensation commensurate with the level of training required for the role. One participant noted that they did not have an educational background in mental health or PSO, which made accessing relevant funding and clinical practice guidelines more challenging. Another participant who had PSO experience but was new to their leadership position explained that they had to complete significant foundational work to address program weaknesses in order to elevate the program to standards they deemed appropriate. 

Directors who were given more control by health authorities recounted acting as an intermediary or buffer in the face of high patient-to-clinician ratios and inconsistent support for activities that facilitate quality improvement, e.g., continuing education or quality assurance projects. Participants described that, depending on the extent to which health authorities granted directors control, PSO service directors could address some of the implementation and monitoring barriers commonly arising from political context. Some directors reported facilitating EBP by protecting time during work hours or offering financial coverage for continuing education and by creating tailored reading recommendations in line with their PSO programming. Participants justified this in saying that these activities not only facilitate clinicians’ services to be evidence-based but also sparked the curiosity and engagement necessary to sustain the long-term emotional demands of providing PSO services. Some participants explained that, although they wanted to create these opportunities for their clinicians, job descriptions were often too narrow to allow for this, and their autonomy as directors was ultimately limited.

When participants were given greater autonomy by health authorities, they assumed that it was due to the high performance metrics of their program relative to other programs, whereas those who perceived restricted autonomy thought it was related to poorer quantitative performance of the program, relatively speaking. Certain participants believed that the only way for their PSO programs to be in the position to monitor their own services would be to create new positions for which the job description includes quality assurance projects, including program evaluation in terms of monitoring quantity, as well as quality of services, including patient-reported outcomes—which reportedly was not possible in most cases.

#### 3.5.2. Internal Communication

Participants stressed the importance of inter- and multidisciplinary connections to communicate about the implementation of PSO services. This was fostered through protected time for regular meetings and events, such as lunch-and-learns. While the extent of such connection seemed to vary widely, participants explained this communication facilitated that patients received the appropriate services, increased the cooperative sharing of evidence-based PSO knowledge, and fostered collaboration on various forms of quality assurance projects and other service monitoring tasks. Participants reported that protected time for internal communication facilitated distress screening, triage and referral, and knowledge of available programming. Several participants mentioned that multidisciplinary meetings helped staff understand each other’s areas of oncology specialization and assign tasks in alignment with the stepped care model. One PSO program had nurses who assisted with psychosocial symptom management. Participants explained that all staff involved in triage and referrals should be aware of available services and when these services are indicated for a specific patient according to the stepped care model as well as which clinician would be best for a patient’s specific PSO needs. Poor internal communication was associated with issues in implementing distress screening as well as triage and referral. 

Participants also described being able to tailor therapeutic interventions and provide more holistic PSO care via internal communication, which was reported to have improved the social atmosphere within PSO programs. Some participants said that, when clinicians shared what worked with a particular patient, oncology teams could develop a clearer direction for a patient’s cancer care from a multidisciplinary perspective. One participant expressed: “When you have a multidisciplinary team, you can see and evaluate and get input from different perspectives so that your lens isn’t myopic—it’s broad”. Several participants described that, without this team-based approach, PSO clinicians experienced low morale and diminished sensitivity to patient needs, partly due to a general feeling of disconnection and isolation. Reluctance of individual clinicians regarding collaboration and cooperation was perceived as detrimental to the effective implementation of evidence-based PSO services.

#### 3.5.3. External Affiliations

Participants described that affiliations with teaching hospitals or universities helped improve PSO services as their program attracted student talent and graduates, was more likely to have formal training protocols in place, and had greater access to research, e.g., the university affiliation ensured clinicians had access to scientific databases and even assistance from librarians for literature searches. While university affiliations could not entirely compensate for the limitations that the political context imposed on cooperative sharing, it did stimulate internal communication about EBP by connecting PSO service providers with specialists higher in scientific literacy who have accessed and interpreted more original research in PSO. Collectively, this was reported to have heightened interest, motivation, and passion for EBP in clinicians. It also increased the likelihood of stakeholder buy-in due to having the capacity to delineate and make the case for the EBP of PSO care.

Moreover, these affiliations increased research collaboration and quality assurance projects involving client feedback and program evaluation. While most PSO clinicians were reportedly not provided with time to act as primary investigators, they sometimes collaborated on study design, project proposals, or participated in data collection as key stakeholders. Participants shared this was more likely to be permitted if such collaboration was included as part of clinicians’ job descriptions. In conclusion, it was ultimately remarked that protected time for internal cooperation and external collaboration was mutually beneficial in building the PSO scientific database and increasing opportunities for PSO programs and clinicians to maintain an EBP.

### 3.6. Economic Context

Participants outlined two main funding models: core funding and “per patient” funding. Core-funded programs had consistent budgets that did not vary annually and were generally allocated to create permanent full-time equivalent positions. Nevertheless, participants shared concerns that frequent turnover among health authority figures created barriers, such as delays in funding for PSO services, caused funding cuts for quality assurance projects, even for PSO programs embedded in teaching hospitals with research mandates, and led to positions remaining unstaffed for extended durations. In contrast to core-funded programs, PSO programs funded on a “per patient” basis provided annual reports on the number of patients served and the type of medical treatments patients received, which informed funding decisions for the following year.

One concern regarding both these funding models included rigid allocation conditions restricting the autonomy of PSO program directors in growing their PSO program services. For example, given that funding is allocated almost exclusively for clinicians’ full-time salaries, a director might not be able to fund administrative assistance or quality assurance projects—even if this would facilitate the implementation and monitoring of evidence-based PSO services. Thus, services or activities of PSO programs that are more tangible to addressing patient volume were difficult to put in place without funding from alternative sources, which was more likely to lead to temporary solutions based on “soft money”. Such rigidity to funding allocation presents an obstacle for programs trying to expand or refine their services.

Participants expressed strong concerns that “per patient” funding models lead to inequitable PSO service access depending on the type of medical treatment patients received and the number of patients serviced by each PSO program. PSO programs with fewer patients were more likely to have funding cuts in subsequent years, which was a detriment to rural providers given that the majority of provincial resources were reportedly aggregated in urban regions. Regarding medical treatments, participants reported that more funding is provided for patients receiving systemic and chemotherapy treatment, less funding is provided for radiation therapy, and virtually no funding is provided for surgical patients, patients receiving hormonal treatments, and patients in the survivorship phase. This is described by one participant, who stated:

“[Provincial Cancer Care Organization] give us funds for each patient receiving radiation or systemic therapy. Our program also has patients that only go the surgical route and are not eligible for our services because we don’t get any funding from that activity. Patients are also only eligible for support up to one year after the end of their treatment. So, once it comes to the survivorship or even the bereavement phases, we don’t tend to get involved with those patients. We just don’t have the capacity”.

Another participant shared that funding was unavailable for cancer patients in the survivorship phase:

“Our mandate here is to only see patients being treated. So as soon as the patient is done [their medical] treatment—we’re supposed to end our care. But she [lead clinician] finds that that’s really when they need the most help, when they’re supposed to go back to normal, they’re just kind of left to their own, they’re not followed by anyone”.

Participants reported that family and caregivers are allies in offering support to patients and also have PSO needs, which were excluded from “per patient” models of funding. Based on a holistic approach to PSO, participants expressed that family members and caregivers should also have access to PSO services—yet often do not receive services no matter the funding model. Participants further shared that patients may benefit from support for tangible needs, such as food, accommodation, and transportation. One example of such support was the provision of “comfort funds” for housing family members when long-term hospitalization was required far from home.

Participants highlighted that two variable financial resources could be procured: donations and funding accessed through specific requests. Donations from individual donors were universally accepted by PSO programs. Not-for-profit community sites tended to rely entirely on these donations. Participants also described being able to apply for funding through competitive grant applications for programming expansion or contract positions for specialized services, such as helping underserved populations. Participants were concerned by the potential discontinuation of services that solely rely on this type of funding. PSO programs with more external affiliations reported having more opportunities to apply for grant funding.

### 3.7. Geographic Context

An additional challenge for EBP and generally for the delivery of PSO services in rural areas was the distance between patients and PSO services as well as between clinicians and training opportunities. Higher travel costs and travel times as well as limited public transit options presented challenges to in-person care, while poor internet connection and low digital literacy were described as barriers for telehealth services. Due to having fewer patients, participants reported running fewer group services, which are less costly than individual sessions. Living in rural areas made it difficult for clinicians to remain science-informed and to pursue specialization in PSO, which negatively impacted the availability of specialized services in rural areas. Similarly, participants expressed having to hire applicants that did not meet minimum educational or experience criteria. In addition to these challenges related to sheer physical distance, participants expressed that “per patient” funding models resulted in diminished financial resources. More positions were part-time, which led to dual reporting where clinicians worked at multiple sites and/or had multiple managers. Participants noted that few resources were available for continuing education, and leadership positions covered vast geographic areas and professional disciplines. One participant disclosed that being the only oncology director for their entire province made it difficult to adequately support their staff in discipline-specific areas. Collectively, these challenges were described as barriers to offering equitable PSO services in rural areas.

Participants also reported efforts to minimize the impact of these barriers to rural PSO services. One participant described that their oncology team consistently connected to coordinate patients’ oncology appointments to occur on the same day. Another participant remarked that the COVID-19 pandemic accelerated their transition to virtual service delivery, which reportedly increased service visibility and the likelihood that patients would access services when the timing was right for them. Telehealth was mentioned as a key solution, enabling patients to access more specialized PSO services not typically available in their area. Similarly, another participant with a rural PSO program asked their clinicians to participate in online grand rounds and co-facilitate virtual groups, which increased access to peer consultation and provided opportunities to further specialize in PSO. In summary, considerate scheduling and facilitative technologies made PSO care more accessible in rural areas. 

## 4. Discussion

The goal of the current study was to examine the perspective of Canadian PSO service directors regarding (a) how EBP in PSO is being implemented in supportive cancer care for adults and their families and how the service quality is monitored, and (b) the barriers and facilitators of evidence-based PSO services. Service directors identified some evidence-based practices in all areas of their PSO programs, including in the screening of patients, intervention delivery, and monitoring of care. Each participant identified challenges to implementing EBP in PSO services, with insufficient funding and protected time being consistently described as major detriments to ensuring clinicians remain science-informed and patient needs are met. Each one reported various approaches attempting to overcome these challenges. 

The major themes identified in this study speak to structural barriers creating a feedback loop system that constrains PSO programs’ efforts to provide evidence-based services. For example, program evaluation based solely on number of clients and wait times results in limited funding. This disincentivizes non-direct service provision activities, such as internal communication and sharing of EBP knowledge among PSO colleagues, which could maximize the effective use of resources by facilitating the stepped care model. At the same time, barriers such as limited data collection mandates, lack of affiliation with universities, and high patient load hinder the initiation of research projects and the engagement in the generation of knowledge that could transform the terms by which the program is evaluated. The following three sections discuss the evidence-based practices and respective barriers and facilitators for each of the three major themes identified in response to research question 1.

### 4.1. Screening for Distress and Referral to PSO Services

**Figure 1:**
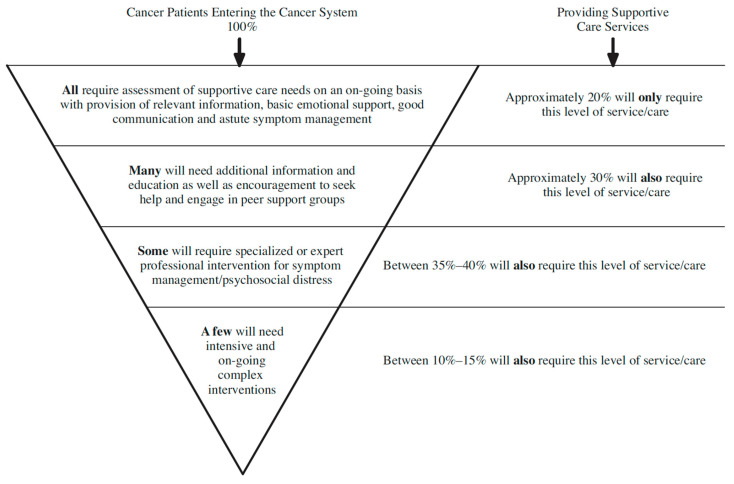
Psychosocial oncology services and estimated proportions of patients requiring each level of care [33] (p. 12, figure reprinted with permission from the *Canadian Oncology Nursing Journal*).

Participants emphasized that the delivery of evidence-based PSO services depends on the accurate identification of patients in distress and the timely referral to appropriate services, which is in line with the existing scientific evidence. The initial and repeated use of distress screening tools is essential for triaging and referring patients to appropriate levels of care at diagnosis throughout treatment and during follow-up care [28]. Directors expressed that PSO programs have a responsibility to facilitate an effective referral process by a) being knowledgeable about available internal and external resources as well as their acceptance criteria and b) ensuring patients are informed in a timely manner to maximize the uptake of appropriate services. Participants explained that services such as psychoeducation, symptom screening, and group interventions should first be prescribed, whereas more resources-intensive interventions, such as individual sessions, would be offered should the former interventions be unsuccessful in managing cancer-related psychosocial problems. This is in accordance with the stepped care model, which Smith and Darling (2015) described as a gold standard for directing the efficient delivery of appropriate care, with simpler interventions being administered at first and moving to more intensive interventions when a good outcome cannot be achieved [29,30]. The application of this model to PSO has been endorsed by various stakeholders, including the Canadian Partnership against Cancer [31], the Canadian Psychosocial Oncology Association [10], and Cancer Care Ontario [32]. There is ubiquitous agreement that all patients with cancer require an assessment of their supportive care needs, while it is proposed that for about 20% of patients it will be sufficient to receive only the most basic support, that 30% will need additional support, such as psychoeducation or peer support groups, that professional interventions to manage psychosocial distress and other cancer-related symptoms will be required for another 35% to 40%, and that 10% to 15% of patients will need the most intensive interventions as presented in Figure 1 [33].

Our research identified inconsistencies in distress screening, cut-off score implementation, patient eligibility based on their medical treatment modality, and service availability in rural areas as systemic barriers to the provision of evidence-based PSO care. All participants except for one reported that some variation in initial distress screening was part of their PSO program’s operating procedures. However, some participants indicated discrepancies in applying this standard due to the real or perceived lack of available follow-up care [28]. Repeated distress screening was rarely implemented, which is consistent with previously documented concerns [34] regarding inconsistent distress monitoring where patients who met the evidence-based cut-off score during initial screening and patients whose distress levels changed over time were not referred to appropriate services. Frequent changes in the availability of internal and/or external PSO services and in respective eligibility criteria as well as low awareness of service availability among certain health providers resulted in patients’ limited awareness of existing resources.

Furthermore, “per patient” based on treatment modality led to offering PSO services to patients receiving certain medical treatments (i.e., systemic and chemotherapy), while no services or only limited services could be offered to patients undergoing other medical regimes (i.e., radiation therapy, surgical patients, hormonal treatments). These models of funding also excluded patients in the survivorship phase and families/caregivers from PSO services. The denial of services to certain patient groups, such as those in the survivorship and bereavement stages, is at odds with recent literature highlighting the importance of addressing the psychosocial needs of the growing survivorship cohort [35] and of holistically addressing family units and caregiver contexts [36]. Participants suggested that the discrepancy between best practice recommendations and the clinical reality is at least partially driven by the fact that health authorities regard PSO as a “nonessential dimension of cancer care”, which is supported by prior research reports [14] and affects the funding allocation to PSO services. Our findings align with previously voiced concerns that many patients who would benefit from PSO care are not receiving it [37].

Participants identified consistent screening standards, digital screening procedures, designated administrative positions, and internal communication as facilitators for distress screening and triage. This is in line with three steps previously put forth to facilitate the Canada-wide implementation of repeated distress screening [32]: (a) establish national progress monitoring standards; (b) raise stakeholder awareness of repeated screening as an EBP standard of care; and (c) secure resources for this. Digital screening procedures can both reduce the misidentification of distress by flagging patients in need of services based on evidence-based, pre-determined cut-off scores, such as reported in the “Pan Canadian Practice Guideline: Screening, Assessment and Care of Psychosocial Distress, Depression, and Anxiety in Adults with Cancer” [38]. Digital screening procedures can also reduce costs associated with manually administering initial and repeat distress screening tools, gather relevant outcome data of patient distress over time, and document the number of patients above the cut-off score who cannot be offered services [31]. People with designated roles, such as administrative positions, resource counsellors, program coordinators, or patient navigators, are all people who helped PSO programs acquire knowledge of available programming and sources, increasing the visibility of services and orienting patients to available services based on their individual preferences before matching them with a service.

Protected time for internal communication facilitated both implementing evidence-based PSO services and monitoring them. Internal communication helped with service delivery and monitoring by increasing clinician knowledge of available programming (especially in the absence of a person with this designated role), facilitated triaging patients to the best available services, and helped clinicians to assign tasks in accordance with the stepped care model. Collaborative sharing was also said to help improve the effective delivery of evidence-based PSO services by increasing a clinician’s involvement with psychosocial symptom management, developing clearer directions for patient care and sensitivity to their needs, as well as through an improved social atmosphere and increased clinician morale. Internal communication and telehealth technologies reduced travel for patients at rural sites through better coordination of all oncology services. Rodin (2018) posits that patients’ perceived support from their healthcare team and the healthcare system more broadly can have a protective effect via increased feelings of validation and safety, regulating painful emotions, greater patient’s self-efficacy, and participation in the decision making process for their care [14].

### 4.2. Delivery of Evidence-Based PSO Services

The delivery of evidence-based PSO services is closely interwoven with clinicians’ acquisition and maintenance of the respective expertise. Wide variations in onboarding training protocols may be a barrier to clinicians uniformly acquiring PSO-specific knowledge when joining the team. Since clinicians arrive at PSO programs with various levels of relevant expertise and experience, well-designed and periodically updated onboarding training protocols that respond to differing training needs and are based on the newest research evidence would be a unique opportunity to ensure all team members acquire the basic knowledge on EBP in PSO. However, it is unrealistic to put this solely on the shoulders of PSO directors. It seems to be a wise investment if health authorities would support the development and delivery of such training opportunities as a joint initiative across provinces.

Participants also reported various levels of being able to support clinician efforts to maintain their PSO knowledge through professional activities including but not limited to supervision and mentorship, journal clubs and communities of practice, peer and case consultations, guest speakers, and external training events. Participants’ concerns were centered on having only minimal and inconsistent health authority support for PSO knowledge acquisition and maintenance activities. Related barriers included lack of access to research databases, limited and inconsistent support for continuing education, narrow definition of performance, as well as restricted job descriptions.

Participants further remarked that clinicians relied more on synthesized research summaries, including recommendations for clinical practice, than on original research studies. However, the utility of clinical practice guidelines that were developed a decade or longer ago is questionable given that the latest research evidence has not been integrated in them. Provincial as well as federal healthcare decision makers would be well advised to invest in the updating of high-quality practice guidelines in PSO as an important means to facilitate EBP.

Facilitators to clinicians acquiring and maintaining a working knowledge of EBP in PSO were almost entirely related to the social context, including the pursuit of a directorial vision, internal communication, and external affiliations. To the extent directors were given control, they attempted to provide protected time during working hours and/or financial support for continuing education and cooperative sharing of EBP within. Directors believed that time for such activities sparked the curiosity and engagement necessary to sustain the long-term emotional demands of providing PSO services. Participants who worked at PSO programs with university affiliations described their onboarding training protocols as comprehensive, evidence-based, and periodically reviewed. They also reported these affiliations increased clinician connection with specialists (i.e., via visiting speakers, conferences or symposiums, and research collaboration), stimulated discussions about latest evidence, and heighted clinicians’ interest and motivation in their clinical work. Thus, it seems beneficial for health authorities to recognize that time for collaborative sharing and internal communication are facilitative to EBP in PSO.

### 4.3. Monitoring of Psychosocial Oncology Services

The American and the Canadian Psychological Association’s definitions of evidence-based practice [4,5,6,7] state that EBP includes the monitoring of the treatments provided, and our study participants stressed the importance of gathering patient-reported outcome data to monitor and improve the quality of PSO interventions and not solely focus on quantitative data, such as number of patients and sessions or average patient wait time. Such a broader perspective is also in line with recommendations from the Canadian Partnership against Cancer [31], a government-funded, independent organization aiming to facilitate cancer control in Canada. Yet, in many PSO programs, only the collection of the latter data (e.g., number of patients seen) was mandated and financially supported by healthcare decision makers. Key barriers to monitoring outcomes included funding cuts for such projects even at teaching hospitals, a lack of autonomy to gather more diversified types of data, and a general lack of control to allocate resources to technological and administrative facilitators. These are clear obstacles regarding EBP in PSO, and participants were painfully aware that collecting more pertinent data would be key to demonstrating the value of PSO services and successful advocacy with financial decision makers. The lack of data on outcomes and patient needs impedes efforts to justify resource requests for collecting those data and providing better services, meaningfully addressing systemic PSO issues. If health authorities want to meet their targets for greater efficiency of PSO services, it seems well advised to move beyond gathering a quite restricted range of quantitative data and towards facilitating the assessment of service quality indicators and acknowledging the value of protected time for focussing on program evaluation and advancement. Technology might already make it possible to extract some additional relevant data at treatment centres that have implemented initial and/or repeated distress screening throughout the illness trajectory. One could, for example, determine the number of patients who should have been offered PSO given their distress score above an evidence-based cut-off score but did not receive a service offer because the PSO program was only able to provide services to patients with the highest distress scores. Other valuable data in the service of quality improvement could be gathered by tracking the uptake of group programming relative to spaces available in a group. Such data have the potential to identify program-specific areas of improvement. It should also be noted that some resources exist for PSO programs to self-evaluate and develop their services [14] in the absence of clear direction from health authorities.

### 4.4. Limitations

Participants representing thirteen unique PSO sites described three categories of service providers excluding private practices: PSO programs within tertiary care settings, community agencies, and cancer centres, the latter of which were named “community oncology programs” elsewhere [10]. Because our ethics protocol required transcripts to be anonymized to keep participants’ identity confidential, it was not possible to compare PSO services by site type. Moreover, the wide variation in program structures each embedded in different political, economic, social, and geographical contexts means that our conclusions are not indicative of each PSO program’s reality. Certain health authorities have called attention to research challenges associated with the lack of standardized program structures [32], indicating that a facilitative attitude should examine individual PSO services as unique entities within an overarching PSO system. Another limitation is that the extent to which PSO programs implemented lower-intensity interventions first, such as psychoeducation and group programming, seemed to vary from program to program; however, this was not explicitly inquired about.

For transferability reasons, future research studies might gather more detailed information about the contexts of each site so readers can better assess how findings are relevant to certain types of PSO programs. Important contextual data might include health authorities and directorial reporting structures, as well as the funding models that PSO programs currently have and had in the recent past. It might also be helpful to examine in more detail PSO program processes for initial and repeated distress screening, triage and referral, the use of digital screening and administrative positions, and whether patients are being offered and are accepting external referrals when PSO programs are unable to offer them services internally.

## 5. Conclusions

Directors of PSO services across Canada indicated that the PSO system is struggling to meet an enormous and growing demand for psychosocial care for cancer patients and their families. Evidence-based recommendations that would likely see a return on investment at a systemic level—notably a stepped care approach with increased use of electronic screening systems and improved administrative processes for ongoing patient triage—have not been widely implemented yet. Moreover, electronic tools could also facilitate gathering patient-reported outcome data that are needed to monitor and improve service quality. However, technology alone is not solving the problem of healthcare professionals being expected to deliver evidence-based PSO care to an ever-growing patient population while having caseloads that prevent them from learning about and implementing EBP in PSO services. Despite the inherent reality of limited resources, protected time is required for the acquisition and maintenance of evidence-based PSO knowledge. It would also seem beneficial if health authorities at provincial or federal level would pool resources to support the development and maintenance of training programs that could be used countrywide. At the same time, healthcare professionals need patient–clinician ratios that allow time for continuing education, for knowledge exchange about EBP, to monitor outcomes, and to collaboratively use those data to improve the quality and quantity of services provided. Ensuring PSO services are evidence-based cannot only rest on the shoulders of PSO directors but necessitates more support from the part of health authorities. Partnering with researchers would be one avenue towards gathering valuable information and evidence that could be used when advocating for the allocation of sufficient resources to implement EBPs. Granting PSO directors more autonomy may help each individual PSO program in meaningfully addressing unique barriers to evidence-based PSO care by allowing them to decide where money and time are most needed. Fostering connections among members of the PSO team as well as with other healthcare professionals involved in cancer care at a given institution could be beneficial to creating a work context better equipped to meet the increasing demands for psychosocial care and a culture within the PSO program that is more resilient to these pressures. Education and advocacy are essential to shift current dichotomous attitudes about quality versus quantity of services towards recognizing their inherent interconnection and interdependence.

## Figures and Tables

**Table 1:** Sociodemographic characteristics of participants (*N* = 16).

	Relative Frequency in % ^1^	*N*
Gender		
Female	62	10
Male	38	6
Age Range (in years)		
25–34	13	2
35–44	25	4
45–54	44	7
55–64	6	1
65+	13	2
Career Stage (in years)		
0–7	13	2
8–14	25	4
15+	62	10
Province		
Quebec	25	4
Ontario	31	5
Manitoba	13	2
Alberta	6	1
New Brunswick	6	1
Nova Scotia	6	1
British Columbia	13	2
Highest Degree		
Bachelor	6	1
Masters	56	9
Doctorate	13	2
Post Doctorate	19	3
Medical Degree	6	1
Profession		
Social Work	38	6
Psychology	31	5
Psychiatry	6	1
Nursing	6	1
Other ^2^	19	3

^1^ Percentages may not total 100 due to rounding. ^2^ This category includes kinesiology, occupational therapy, and administration.

**Table 2:** Overview of themes and subthemes identified for each research question.

Themes and Subthemes	Illustrative Summary of Participant Responses to Research Questions
	**Research question 1: How are evidence-based practices in psychosocial oncology implemented in clinical care and how is the service quality monitored?**
Screening for distress and referral to PSO services.	
Initial and Repeated Distress Screening	Assessing patient distress from first point of contact and over time using validated tools to accurately identify patients in need of PSO services. Discrepancies identified between intended operating procedures and the reality of implementing such tools.
Triage and Referral Systems	Description of decision making processes for offering PSO services or to refer patient elsewhere. Knowledge of internal and external resources described as necessary for offering best possible follow-up care. Ideally, patients are informed about their choices and provided opportunity to express their individual preferences.
Administration and Technology	Technology and administrative positions, such as resource counsellors, described as key facilitators to efficient distress screening, triage, and referral process.
Delivery of evidence-based PSO services.	
Therapeutic Interventions	Interventions designed specifically for oncology patients and other evidence-based psychosocial interventions commonly used in PSO service delivery.
Credentials and prior professional experience	Elaboration on the ideal educational requirements for a PSO clinician. Related prior professional experience viewed as favorable for joining the PSO services team.
Onboarding Training Protocols	The described training protocols for incoming team members as well as for students differ in their comprehensiveness.
Ongoing access to research and training	Research summaries, such as clinical practice guidelines, were preferred over original research reports and viewed as facilitators of evidence-based practice. Limited access to scientific databases and limited time for research-related activities were described as barriers. Further activities to support EBP included supervision and mentorship, journal clubs and communities of practice, peer and case consultations, conferences or symposiums, and research collaboration.
Specialization and attitude of curiosity	Specialization, e.g., by cancer site or population attributes, described as advantageous for addressing patient needs and useful for clinicians’ wellbeing and for research agendas.
Monitoring of PSO services.	
Patient Feedback	Monitoring of PSO service outcomes through soliciting patient feedback about their experience described as necessity to ensure effectiveness and to improve future care.
Requirements of licensing bodies and performance reviews	Clinician membership with regulatory colleges viewed as means to fostering evidence-based practices, e.g., through continuing education requirements. Performance evaluations used by some programs for constructive feedback and to collaboratively set professional goals.
Program Evaluation	Participants reported having PSO program evaluations, even if infrequent and informal. They expressed reporting on quantity metrics, such as wait times and number of patients served.
Research and Quality Assurance Projects	Research completed to evaluate program efficiency and effectiveness; also useful for PSO advocacy and to improve quality of care provided, yet mainly led by external principal investigators.
	**Research question 2: What are the barriers and facilitators of evidence-based practice in psychosocial oncology services?**
Political Context	Health authorities, positioned at the top of vertical hierarchies, and certain directors largely determined how resources were used in PSO programs. Health authorities’ prioritization of quantity over quality, as evidenced by the type of data gathering required of service providers, was believed to have contributed to high patient-to-clinician ratios, which in turn was described as a barrier for clinicians to maintain knowledge about EBP in PSO.
Social Context	
Directorial Vision	As directors of PSO programs, participants reported being uniquely positioned to improve implementation and monitoring of EBP. This was only true to the extent they were given control by health authorities to do so and if directors were specialized in PSO.
Internal Communication	Internal communication facilitates increased awareness of available PSO services and improves client-centered care, whereas a lack thereof has negative impact on work environments and PSO service implementation.
External Affiliations	University affiliations described as a facilitator of EBP in PSO, attracting trainees, better research and teaching integration, and greater stakeholder buy-in to support PSO services.
Economic Context	Source and consistency of funding had impacts on implementing and monitoring PSO services. Concerns were expressed regarding “per patient” models, which led to inequitable access across the cancer trajectory, and, overall, having rigid conditions for funding allocation was viewed as detriment to meet observed needs in patients within a timely manner.
Geographical Context	Geography reported as a barrier for patient access to PSO services, including less or no group programming, as well as clinicians accessing opportunities to specialize. Increased internal communication described as beneficial for best possible service provision despite limitations.

## Data Availability

The data presented in this study are available on request from the corresponding author. The data are not publicly available to keep the identity of our participants confidential. Anonymized data will be shared under the condition that the guidelines of McGill University’s Research Ethics Board are respected.

## Supplementary Materials

The following supporting information can be downloaded at: https://www.mdpi.com/article/10.3390/curroncol30040303/s1, Supplementary S1: Semi-Structured Interview Guide; Table S1: Intercoder Reliability Data Table.

## References

[1] American Psychological Association (2017). Ethical Principles for Psychologists and Code of Conduct.

[2] Canadian Psychological Association (2017). Canadian Code of Ethics for Psychologists.

[3] Wiener L., Viola A., Koretski J., Perper E.D., Patenaude A.F. (2015). Pediatric Psycho-Oncology Care: Standards, Guidelines, and Consensus Reports: Pediatric Psychosocial Standards, Guidelines, and Consensus Reports. Psychooncology.

[4] American Psychological Association (2006). Evidence-Based Practice in Psychology. Am. Psychol..

[5] Dozois D.J. (2014). The CPA Presidential Task Force on Evidence-Based Practice of Psychological Treatments. Can. Psychol..

[6] Gaudiano B.A., Miller I.W. (2013). The Evidence-Based Practice of Psychotherapy: Facing the Challenges That Lie Ahead. Clin. Psychol. Rev..

[7] Kazdin A.E. (2008). Evidence-Based Treatment and Practice: New Opportunities to Bridge Clinical Research and Practice, Enhance the Knowledge Base, and Improve Patient Care. Am. Psychol..

[8] Dobson K. (2016). Clinical Psychology in Canada: Challenges and Opportunities. Can. Psychol. Can..

[9] Ionita G., Fitzpatrick M. (2014). Bringing Science to Clinical Practice: A Canadian Survey of Psychological Practice and Usage of Progress Monitoring Measures. Can. Psychol. Can..

[10] Canadian Association of Psychosocial Oncology (2010). Standards of Psychosocial Health Services for Persons with Cancer and Their Families.

[11] Adler N.E., Page A., Institute of Medicine (2008). Cancer Care for the Whole Patient: Meeting Psychosocial Health Needs.

[12] Breitbart W., Butow P., Jacobsen P., Lam W., Lazenby M., Loscalzo M. (2021). Psycho-Oncology.

[13] Jacobsen P.B., Wagner L.I. (2012). A New Quality Standard: The Integration of Psychosocial Care Into Routine Cancer Care. J. Clin. Oncol..

[14] Rodin G. (2018). From Evidence to Implementation: The Global Challenge for Psychosocial Oncology. Psychooncology.

[15] Hack T.F., Carlson L., Butler L., Degner L.F., Jakulj F., Pickles T., Dean Ruether J., Weir L. (2011). Facilitating the Implementation of Empirically Valid Interventions in Psychosocial Oncology and Supportive Care. Support. Care Cancer.

[16] Coleman N., Hession N., Connolly A. (2011). Psycho-Oncology Best Practice Guidelines and a Service Perspective: Conceptualising the Fit and towards Bridging the Gap. Ir. J. Psychol..

[17] Turner J., Zapart S., Pedersen K., Rankin N., Luxford K., Fletcher J. (2005). Clinical Practice Guidelines for the Psychosocial Care of Adults with Cancer. Psychooncology.

[18] Turner J., Rankin N., Lam W.W.T., Breitbart W., Butow P., Jacobsen P., Lam W., Lazenby M., Loscalzo M. (2021). Implementation of Clinical Practice Guidelines for Psychosocial Cancer Care. Psycho-Oncology.

[19] Gergen K.J. (2015). Relational Being: Beyond Self and Community.

[20] Stake R.E. (1995). The Art of Case Study Research.

[21] Levitt H.M., Bamberg M., Creswell J.W., Frost D.M., Josselson R., Suárez-Orozco C. (2018). Journal Article Reporting Standards for Qualitative Primary, Qualitative Meta-Analytic, and Mixed Methods Research in Psychology: The APA Publications and Communications Board Task Force Report. Am. Psychol..

[22] Patton M.Q. (2014). Qualitative Research & Evaluation Methods.

[23] Braun V., Clarke V. (2006). Using Thematic Analysis in Psychology. Qual. Res. Psychol..

[24] Eccleston P., Werneke U., Armon K., Stephenson T., MacFaul R. (2001). Accounting for Overlap? An Application of Mezzich’s j Statistic to Test Interrater Reliability of Interview Data on Parental Accident and Emergency Attendance. J. Adv. Nurs..

[25] Mezzich J.E., Kraemer H.C., Worthington D.R.L., Coffman G.A. (1981). Assessment of Agreement among Several Raters Formulating Multiple Diagnoses. J. Psychiatr. Res..

[26] Burla L., Knierim B., Barth J., Liewald K., Duetz M., Abel T. (2008). From Text to Codings: Intercoder Reliability Assessment in Qualitative Content Analysis. Nurs. Res..

[27] Landis J.R., Koch G.G. (1997). The Measurement of Observer Agreement for Ranked Data. Biometrics.

[28] Mitchell A.J., Holland J.C., Breitbart W.S., Jacobsen P.B., Loscalzo M.J., McCorkle R., Butow P.N. (2015). Screening and Assessment for Distress. Psycho-Oncology.

[29] Von Korff M., Tiemens B. (2000). Individualized Stepped Care of Chronic Illness. West. J. Med..

[30] Loscalzo M.J., Bultz B.D., Clark K., Jacobsen P.B., Holland J.C., Breitbart W.S., Jacobsen P.B., Loscalzo M.J., McCorkle R., Butow P.N. (2015). Building Supportive Care Programs in a Time of Great Opportunity. Psycho-Oncology.

[31] Canadian Partnership Against Cancer (2012). Screening for Distress, the 6th Vital Sign: A Guide to Implementing Best Practices in Person-Centered Care. Cancer Journey Portf..

[32] Cancer Care Ontario (2018). Recommendations for the Delivery of Psychosocial Oncology Services in Ontario.

[33] Fitch M. (2008). Supportive Care Framework. Can. Oncol. Nurs. J..

[34] Jacobsen P.B. (2009). Promoting Evidence-Based Psychosocial Care for Cancer Patients: Promoting Evidence-Based Psychosocial Care. Psychooncology.

[35] Lustberg M.B., Carlson M., Nekhlyudov L. (2021). Introduction to Special Section: Living with Incurable Cancer: Addressing Gaps in Cancer Survivorship. J. Cancer Surviv. Res. Pract..

[36] Hoge M.A., Roth A.J., Holland J.C., Breitbart W.S., Jacobsen P.B., Loscalzo M.J., McCorkle R., Butow P.N. (2015). Training Psychiatrists and Psychologists in Psycho-Oncology. Psycho-Oncology.

[37] Jacobsen P.B., Shibata D., Siegel E.M., Lee J.-H., Fulp W.J., Alemany C., Abesada-Terk G., Brown R., Cartwright T., Faig D. (2011). Evaluating the Quality of Psychosocial Care in Outpatient Medical Oncology Settings Using Performance Indicators. Psychooncology.

[38] Howell D., Keshavarz H., Esplen M., Hack T., Hamel M., Howes J., Jones J., Li M., Manii D., McLeod D. (2015). A Pan Canadian Practice Guideline: Screening, Assessment and Care of Psychosocial Distress, Depression, and Anxiety in Adults with Cancer.

