# Supplementary material for: Evidence-Based Practice in Psychosocial Oncology from the Perspective of Canadian Service Directors
Source: Curr Oncol. 2023 Apr 1;30(4):3998–4020. doi: 10.3390/curroncol30040303 (PMC10136815; doi:10.3390/curroncol30040303)
Supplement: Supplementary file 1 [file curroncol-30-00303-s001.zip › Supplementary S1_Semi-Structured Interview Guide.pdf]

Semi-Structured Interview Guide

[Note: This interview guide is meant to facilitate open-ended responses from participants. For this reason, actual questions may be slightly different from what are presented here, as well as possibly presented in a different order. Although it is difficult to anticipate what kind of additional follow-up questions may be asked, this guide provides the spirit of the kinds of questions that will be asked.]

PRE-INTERVIEW

a. [Express gratitude to participant].
b. [Review key points of informed consent document including being audio-recorded / present structure of interview / ask if participant has any questions.]
c. [Introductory prompt:] Throughout the interview I will be asking about practitioners, by this I am referring to clinicians working in your program who deliver psychosocial assessment, psychotherapy, and/or counselling, to adults (18+) diagnosed with cancer and their families.

I. OVERLL PROGRAM STRUCTURE

1. I thought we could begin by talking about your program generally. I was wondering how your psychosocial oncology program structured? (Do you have a source of funding and is it consistent? How do you allocate your budget? Are the psychosocial care providers employee's or volunteers?)

II. SPECIFIC QUESTIONS ABOUT PROGRAM

Now I would like to ask you some specific questions about the program, in terms of its policies and standards of care, as well as mechanisms, resources and tools.

2. What are your current policies and standards of psychosocial care? (What informed these standards? How often are these reviewed?)
3. Which mechanisms does your program have in place to facilitate best cancer care? (What resources and tools do your practitioners use?)
4. How do you monitor the quality of care provided to clients across the cancer trajectory from intake to termination? (Do you use any tools to assess the processes and outcomes of care? What do you do with the results of your quality care assessment?)

III. SPECIFIC QUESTIONS ABOUT PRACTITIONERS

Now I would like to ask you about barriers and facilitators to evidence-based practice that practitioners may perceive in your program. [Provide current APA and CPA definition of EBP].

5. What barriers and facilitators do practitioners experience for the delivery of best care?
6. How do your practitioners use scientific evidence to inform their practice? (How confident do practitioners feel evaluating the quality of research? What kind of research do they access?)
7. What *training* and *skills* are practitioners expected to have when hired for POP? (Does your program have a clinical training protocol? Does your program have a research training protocol? How was this training protocol developed?)
8. How are practitioners supported in their pursuit of ongoing research-related education? (What types of ongoing EBP training opportunities are available to practitioners internally and externally? What types of challenges do practitioners experience in accessing these opportunities?)

IV. POST-INTERVIEW

a. [Express gratitude.]
b. [Do you know any other directors who might be interested in participating in this study?]
