# Supplementary material for: Evidence-Based Practice in Psychosocial Oncology from the Perspective of Canadian Service Directors
Source: Curr Oncol. 2023 Apr 1;30(4):3998–4020. doi: 10.3390/curroncol30040303 (PMC10136815; doi:10.3390/curroncol30040303)
Supplement: Supplementary file 1 [file curroncol-30-00303-s001.zip › Table S1_Intercoder reliability Data.pdf]

Intercoder Reliability Data Table

Table 1. Code Occurrence, Agreement Rate, and Nonassignment Frequencies per Code and Rater ($n_{transcripts}$ = 4)

Code	Code Occurrence	Agreement Rate in %	Relative Frequency of not Assigning a Code per Rater	
			Rater 1	Rater 2
Time and availability	16	88	1	1
Education level	12	83	2	0
Money	21	81	4	0
General and oncology-specific experience	10	80	1	1
Conferences	14	79	3	0
Best-practices sources	14	75	3	1
Collaborative work	14	57	1	5
Administrative and technical	11	54	5	0
Cooperative sharing	13	54	2	4
Internal communication	18	50	2	5
Institutional support	10	50	3	1
Professional roles and salaries	13	46	3	4
Chief determinism	12	42	2	4
Client social support	14	29	6	4
Clinical research projects	4	100	0	0
Progress monitoring	3	100	0	0
Connection and isolation	8	88	0	1
Training protocol	7	86	0	1
Coalescence of best-practices	6	83	1	0
Source and consistency of funding	5	80	0	1
Therapeutic interventions	7	71	1	1
Patient-practitioner ratio	9	67	0	3
Distress screening	9	67	1	2
Research accessibility	9	67	2	1
Training opportunities	8	63	1	1
Client feedback	8	63	1	2
Evolution in professional context	9	56	2	3
Autonomy level	8	50	3	2
Program evaluation	8	50	3	2
University affiliation	6	50	0	3
Budget allocation	4	50	1	1
Practitioner reflexivity and flexibility	5	40	1	2
Agreement rate over all codes		65.5	–	–
Interrater reliability for all 32 codes	κ = .64			

Note. Often used codes (≥10) are above, and less used codes (≤10) are below. Codes are sorted according to descending rate of agreement.
